# Radiomic approach to support multidisciplinary tumor board decision-making in locally advanced non-small cell lung cancer

**DOI:** 10.3389/fonc.2025.1713847

**Published:** 2025-12-19

**Authors:** Giulia Pasello, Harel Kotler, Alessandra Ferro, Luca Bergamin, Elena Scagliori, Angela Grassi, Fabio Aiolli, Mattia De Nuzzo, Marco Schiavon, Matteo Sepulcri, Marco Krengli, Valentina Guarneri, Francesca Caumo, Gisella Gennaro

**Affiliations:** 1 Medical Oncology 2, Veneto Institute of Oncology IOV – IRCCS, Padova, Italy; 2 Department of Surgery, Oncology and Gastroenterology, University of Padova, Padova, Italy; 3 Breast Radiology Unit, Veneto Institute of Oncology IOV – IRCCS, Padova, Italy; 4 Department of Mathematics, University of Padova, Padova, Italy; 5 Radiology Unit, Veneto Institute of Oncology IOV – IRCCS, Padova, Italy; 6 Clinical Research Unit, Veneto Institute of Oncology IOV-IRCCS, Padova, Italy; 7 Thoracic Surgery Unit, Department of Cardiac, Thoracic and Vascular Sciences and Public Health, University of Padova, Padova, Italy; 8 Radiotherapy Unit, Veneto Institute of Oncology IOV – IRCCS, Padova, Italy

**Keywords:** locally advanced non-small cell lung cancer, lung cancer, multidisciplinary tumor board, radiomics, stage III non-small cell lung cancer

## Abstract

**Background and objective:**

Selecting the optimal treatment for locally advanced non-small cell lung cancer (LA-NSCLC) is complex and typically requires multidisciplinary tumor board (MTB) evaluation. This study investigated whether machine learning (ML) models trained on MTB decisions could support treatment selection by integrating clinicopathological characteristics with radiomic features from both the primary tumor and mediastinal lymph nodes (LN).

**Materials and methods:**

We retrospectively analyzed patients with LA-NSCLC whose treatments had been decided by an expert MTB. Patients were categorized into three pathways: (A) upfront surgery, (B) neoadjuvant systemic treatment followed by surgery, (C) concurrent chemoradiotherapy. Baseline CT scans were segmented to extract radiomic features from primary tumors and mediastinal LNs. Two ML models were developed based on clinicopathological and radiomic data, using MTB decisions as ground truth: (1) A vs. Rest and (2) B vs. C. Performance was assessed in independent training and test cohorts using the area under the receiver operating characteristic curve (AUC) and accuracy.

**Results:**

In the training cohort, the A vs. Rest achieved an AUC of 0.847 and accuracy of 0.795 with 13 features, while the B vs. C model reached an AUC of 0.740 and accuracy of 0.700 with 9 features. In the test cohort, results remained robust, with an AUC of 0.808 (accuracy 0.700) for A vs. Rest and an AUC of 0.754 (accuracy 0.740) for B vs. C.

**Conclusions:**

ML models combining clinicopathological and radiomic features can reproduce MTB treatment recommendations for LA-NSCLC with good accuracy. This approach may provide decision in settings with limited MTB expertise and promote more consistent treatment allocation.

## 1 Introduction

Lung cancer (LC) is the second most commonly diagnosed cancer and the leading cause of cancer-related mortality worldwide, with high death rates observed in both men and women (1). Non-small cell lung cancer (NSCLC) accounts for approximately 85% of all LC cases, and about 30% of these patients present with locally advanced (LA) disease (stage III according to the eight edition of the TNM classification) at diagnosis. LA-NSCLC is a highly heterogeneous condition, with wide variations in tumor size, local extension, and nodal involvement, making optimal treatment selection a persistent challenge in thoracic oncology (2–4). Although LA-NSCLC is potentially curable, it remains associated with a high tumor burden and poor outcomes, with 5-year survival rates of 36%, 26%, and 13% for stages IIIA, IIIB, and IIIC, respectively (5). This scenario underscores the need for treatment standardization, careful optimization, ideally guided by experienced multidisciplinary teams (6).

Multidisciplinary tumor boards (MTBs) play a crucial role in the management of LA-NSCLC. However, heterogeneity in MTB expertise, case volumes, and access to advanced surgical, radiotherapy, and systemic treatment options can influence the appropriateness of treatment strategies and, ultimately, patient outcomes (7, 8). Moreover, the rapid introduction of novel therapeutic approaches, including targeted agents and immunotherapy in both perioperative and post-chemoradiotherapy (CRT) settings, has improved survival but blurred traditional treatment boundaries, further emphasizing the importance of precise patient stratification to guide personalized care (9–23).

In this evolving landscape, additional prognostic and predictive biomarkers are needed to complement established markers such PD-L1. Inflammatory signatures in blood and tumor samples, tumor immune microenvironment features and radiomic descriptors are increasingly recognized as potential contributors to refining treatment allocation (24, 25). Radiomics, in particular, enables extraction of high-dimensional quantitative features from routine imaging and has shown promise in oncology as a non-invasive biomarker discovery tool (26). In LA-NSCLC, computed tomography (CT)-based radiomic signatures from primary tumors has been explored for predicting occult mediastinal LN involvement (27, 28). Furthermore, preliminary evidence suggests that radiomic features derived directly from LNs may provide even more relevant information than those from the primary tumor alone (29–31). However, LN-based radiomics remains underexplored, and the integration of these features with clinicopathological data has been scarcely investigated.

In this proof-of-concept study, we retrospectively analyzed 176 patients who underwent radical treatment for stage III NSCLC over a 10–year period (2012-2022). We collected clinicopathological, radiological and treatment data, and integrated them with radiomic features extracted from both the primary tumor and mediastinal LNs. Treatment allocation, determined by the local expert MTB, served as the reference standard. Our aim was to explore the feasibility and potential utility of machine learning models trained on these data to reproduce MTB decisions. Such an approach could ultimately provide decision-support tools for centers with limited MTB expertise, thereby promoting more consistent and individualized treatment strategies in LA-NSCLC.

## 2 Materials and methods

### 2.1 Study design and population

This proof-of-concept retrospective observational study included patients with stage III NSCLC consecutively referred to the Thoracic Oncology MTB at the Veneto Institute of Oncology IOV – IRCCS and the University Hospital of Padua (Italy) between November 2012 and July 2022. The study was approved by the Ethics Committee of the IOV-IRCCS (Cod. Int CESC IOV: 2021-89). Written informed consent for data collection, analysis and publication was obtained from all participants in accordance with the regulations of the Italian Data Protection Authority.

**Figure 1:**
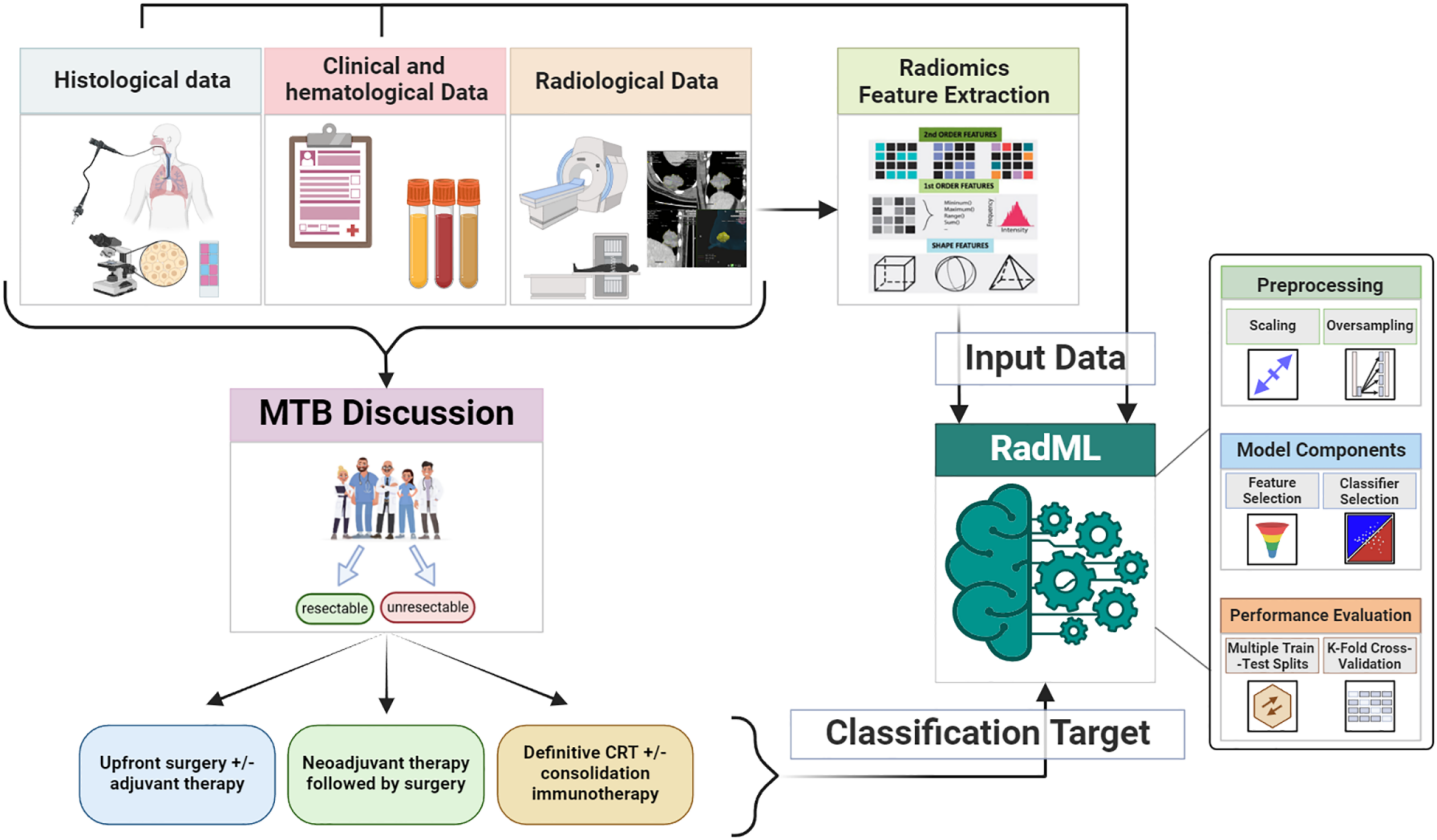
Study scheme. The diagnostic-therapeutic pathway of eligible patients is herein depicted, followed by the radiomics analysis process, from images segmentation to features extraction and data analysis. CRT, chemo-radiotherapy; MTB, multidisciplinary tumor board.

The study scheme is depicted in **Figure 1**.

Inclusion criteria were: (i) histologically confirmed stage III NSCLC; (ii) eligibility for multimodality treatment with radical intent as determined by the MTB; (iii) availability of clinical, hematological, and pathological data; (iv) availability of a baseline total-body CT scan and 18F-fluorodeoxyglucose positron emission tomography (18F-FDG PET-CT); (v) presence of at least one pulmonary target lesion (longest diameter greater than or equal to 10 mm) and -if available- one or two mediastinal LNs that were PET-avid and/or pathologically confirmed as positive; and (vi) a minimum follow-up of 6 months after the completion of radical treatment.

**Figure 2:**
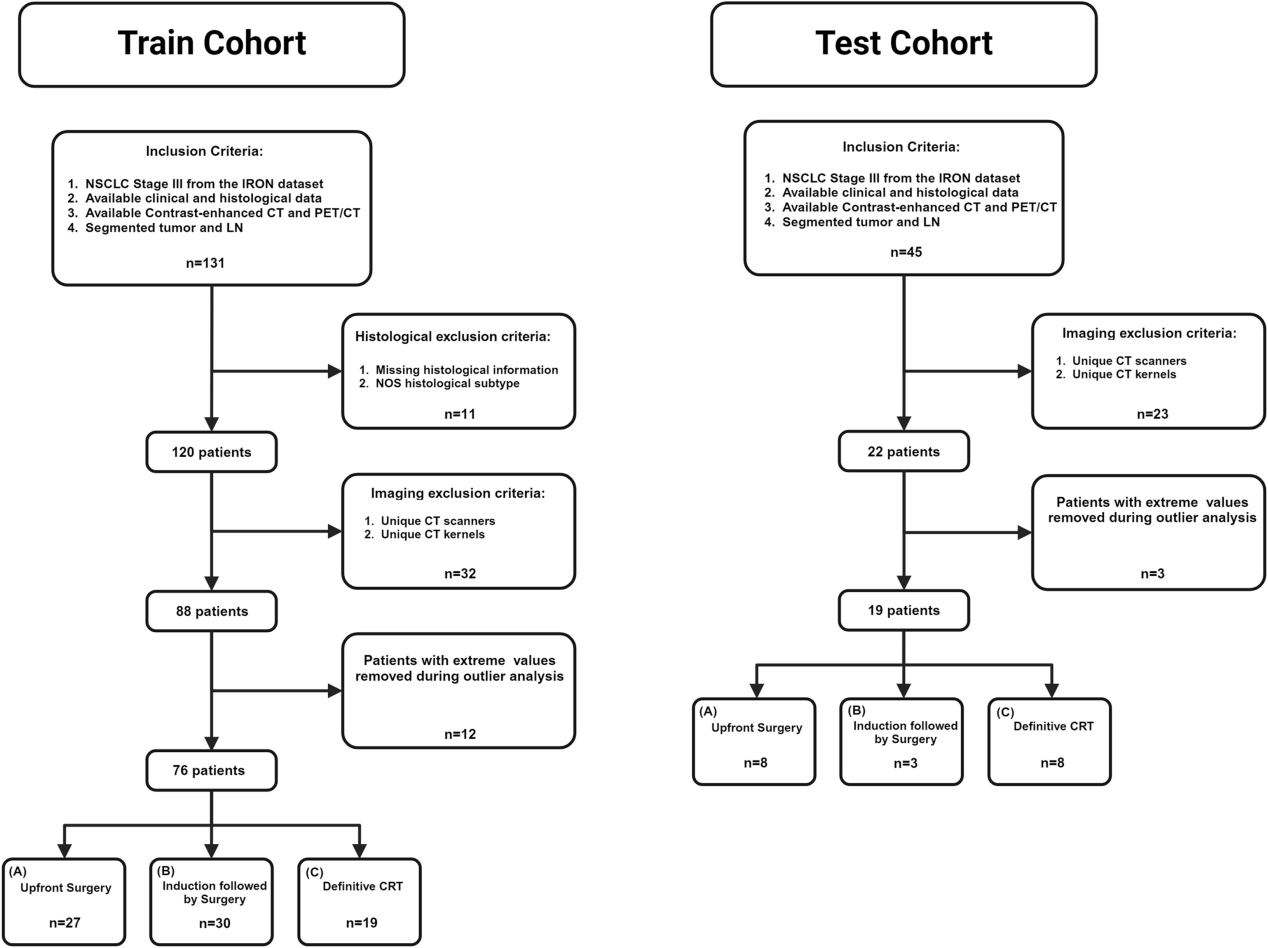
Participant flow diagrams of the train and test datasets. CT, computed tomography; CRT, chemo-radiotherapy; LN, lymph node; NOS, not otherwise specified; Neoadj, neoadjuvant therapy; NSCLC, non-small cell lung cancer; PET/CT, positron emission tomography.

Patients were excluded if their CT images had a slice thickness > 5 mm or were acquired using a single non-replicated CT-scanner model. PET-CT was mandatory to integrate CT-derived LN status. The study population and detailed eligibility criteria are presented in **Figure 2**.

### 2.2 Clinicopathological data and treatment pathways

For each patient, we collected clinicopathological variables including age, sex, Eastern Cooperative Oncology Group Performance Status (ECOG PS), smoking history, tumor histology, molecular profile, PDL1 expression levels, and TNM stage (American Joint Commission on Cancer (AJCC) – 8th edition. The data was anonymized and collected as an electronic case report form.

Three treatment courses with curative intent were defined by the MTB according to clinical guidelines and patient characteristics:

- Course A: upfront surgery, with or without adjuvant systemic therapy.
- Course B: neoadjuvant systemic therapy followed by surgery.
- Course C: concomitant or sequential CRT followed by consolidation immunotherapy when indicated.

Patients receiving radical radiotherapy alone were not included. During the study timeframe, treatment standards evolved: platinum-based ChT remained the backbone in neoadjuvant and adjuvant settings, with immunotherapy gradually introduced in Italy from 2018 (consolidation after CRT) and perioperative chemo-immunotherapy or targeted agents accessible through clinical trials or compassionate programs. Details about the diagnostic-therapeutic pathway were previously reported (32). A reproduction of the decision-making clinical algorithm is summarized in Supplementary **Figure 1**.

To assess the appropriateness of each clinical decision, patient outcomes were analyzed in terms of median relapse-free survival (RFS) and median overall survival (OS) within the intention–to-treat (ITT) population for each treatment course.

### 2.3 Image segmentation and feature extraction

Baseline CT and FDG-PET/CT scans were reviewed for each patient. PET/CT was used to identify pathological mediastinal LNs based on abnormal FDG uptake or histological confirmation, but all segmentations were performed exclusively on the CT images.

Semi-automatic CT-based segmentation of the primary tumors and mediastinal LNs were performed using HealthMyne software v. 13.6 (HealthMyne, Madison, WI) by a chest radiologist with 10 years of experience, in consensus with a thoracic oncologist. Up to four lesions were segmented per patient: one or two primary lung parenchymal lesions and one or two mediastinal LNs. LN selection was based on short-axis >15 mm on CT, PET evidence of pathological uptake, or histological confirmation of involvement. Each patient, therefore, had up to four segmentations: 1–2 lung parenchymal tumors and 1–2 mediastinal LNs. All segmentations were subsequently verified by a senior radiologist with >25 years of experience.

Radiomic features (first-order statistics, shape, and higher-order texture descriptors) were extracted from each CT segmentation. To reduce variability, pre-processing included exclusion of zero-variance features, harmonization across scanners using the NeuroCombat method (33), and removal of extreme outliers (upper and lower 10% of the interquartile range).

### 2.4 Machine learning pipeline: RadiomiX

Radiomic and clinicopathological features were analyzed using RadiomiX, an automated machine learning framework designed for radiomic analysis. RadiomiX systematically evaluates multiple combinations of feature selection strategies and classifiers to identify robust and reproducible models while mitigating the risk of overfitting (34).

The classification was structured in two sequential tasks:

1. Course A vs Courses B+C (upfront surgery vs. multimodality treatment).

2. Course B vs. Course C (f neoadjuvant therapy plus surgery vs. CRT-based treatment).

For each task, RadiomiX pipeline evaluated 532 model combinations. The modelling workflow consisted of 10 randomized train–test splits, each preserving the original class distribution. Within every split, a nested 5-fold cross-validation was applied. All preprocessing steps were executed exclusively within the training folds to avoid information leakage.

Model performance was evaluated using area under the receiver operating characteristic curve (AUC) and accuracy. AUC and accuracy were computed for every folds and then averaged across folds and across all 10 splits to obtain final cross-validated performance estimates. Models achieving a mean AUC and accuracy ≥0.70 were considered well-performing. Among these, the model with the highest mean cross-validated AUC was selected as the final model for each task.

Across the 532 combinations, Minimum Redundancy Maximum Relevance (MRMR) emerged as the best-performing feature selection method for both classification tasks. The top classifiers identified were a Support Vector Machine (SVM) for the Course A vs. Rest and a Random Forest for the Course B vs. C.

Feature stability within the modelling process was assessed by quantifying the recurrence of MRMR-selected features across all 10×5 cross-validation iterations. features retained in the final models consistently ranked among those with the highest selection frequencies, typically appearing in >70% of resampling iterations, indicating good robustness to repeated validation. A detailed summary of feature frequencies and their relationship with model coefficients is provided in the **Supplementary Material**.

Final models were tested on the independent test cohort. To derive stable performance estimates, 100 bootstrap resamplings of the test cohort were performed, and performance metrics were averaged across iterations.

### 2.5 Statistical analysis

Clinical outcomes were analyzed descriptively to contextualize MTB treatment decisions. Relapse-free survival (RFS) was defined from radical surgery or CRT initiation to first recurrence; overall survival (OS) was defined from histological diagnosis to death. Patients without events were censored at last follow-up.

Although the primary endpoint was to replicate MTB treatment decisions, survival outcomes were analyzed across treatment groups as an exploratory check. The aim was to demonstrate that the MTB-based treatment decisions used as reference labels were consistent with clinical outcomes. Kaplan–Meier curves were generated according to treatment groups and model-predicted classes.

All analyses were conducted using Python 3.9.16. The optimized configurations of the models selected for each classification task are detailed in the Supplementary Material. Statistical evaluations were performed using OriginPro 2024 (OriginLab Corporation, Northampton, MA, USA) and MedCalc Statistical version 22.021 (MedCalc Software Ltd, Ostend, Belgium).

## 3 Results

### 3.1 Population description

From the initial ITT population of the train cohort (N = 131), 55 patients were excluded due to missing clinical data or technical reasons (**Figure 2**), leaving 76 patients for analysis. The majority were males (N = 45; 59%), with ECOG PS 0 –1 (N = 73; 96%), and adenocarcinoma histology (N = 55; 72%). Patient characteristics and the clinicopathological features are summarized in **Table 1**. Treatment distribution was: course A (upfront surgery) N = 27 (36%), course B (neoadjuvant therapy followed by surgery) N = 30 (39%), and course C (chemo-radiotherapy, with or without consolidation immunotherapy) N = 19 (25%).

**Table 1:** Patients’ characteristics of the train and test cohorts.

Characteristic	Train cohort N (%)	Test cohort N (%)
Total number of patients	76 (100%)	19 (100%)
Gender		
Male	45 (59.2%)	12 (63.2%)
Female	31 (40.8%)	7 (36.8%)
Age (y)	66.8 ± 9.1	68.6 ± 8.3
ECOG PS		
0	42 (55.3%)	8 (42.1%)
1	33 (43.4%)	11 (57.9%)
2	1 (1.4%)	0 (0.0%)
Stage		
IIIA	43 (56.6%)	9 (47.4%)
IIIB	30 (39.5%)	10 (52.6%)
IIIC	3 (3.9%)	0 (0.0%)
cN		
N0	3 (3.9%)	1 (5.3%)
N1	12 (15.8%)	5 (26.3%)
N2	54 (71.1%)	10 (52.6%)
N3	7 (9.2%)	3 (15.8%)
Histology		
Adenocarcinoma	55 (72.4%)	15 (78.9%)
Squamous cell carcinoma	21 (27.6%)	4 (21.1%)
EGFR status*		
Ex19Del/L858R mutation	7 (12.7%)	2 (13.3%)
Uncommon mutation	5 (9.1%)	2 (13.3%)
Wild type	42 (76.4%)	11 (73.3%)
Missing	1 (1.8%)	0 (0.0%)
ALK status*		
EML4-ALK fusion	2 (3.6%)	2 (13.3%)
Wild type	52 (94.5%)	13 (86.7%)
Missing	1 (1.8%)	0 (0.0%)
PD-L1		
<1%	40 (52.6%)	6 (31.6%)
1–49%	20 (26.3%)	7 (36.8%)
≥50%	16 (21.1%)	6 (31.6%)
Smoking status		
Never	15 (19.7%)	3 (15.8%)
Current	31 (40.8%)	7 (36.8%)
Former	30 (39.5%)	9 (47.4%)
Course of action		
A (upfront surgery)	27 (35.5%)	8 (42.1%)
B (neoadj+surgery)	30 (39.5%)	3 (15.8%)
C (CRT)	19 (25.0%)	8 (42.1%)

*available for 54 out of 55 patients with adenocarcinoma in the Train cohort and for all patients with adenocarcinoma in the Test cohort.

cN, clinical N stage; CRT, chemo-radiotherapy; Ex19Del, exon 19 deletion; Neoadj, neoadjuvant therapy; PD-L1, programmed death-ligand 1.

In the test cohort, 26 of 45 patients were excluded using the same criteria, leaving 19 patients for analysis. Among them, 8 (42%) were assigned to course A, 3 (16%) to course B; and 8 (42%) to course C.

Systemic therapy details for both cohorts are provided in **Table 2**. Notably, a few patients were treated within clinical trials [IMpower030, AEGEAN (35, 36)] or received adjuvant osimertinib through compassionate use programs.

**Table 2:** Systemic therapies administered according to treatment course in the train and test cohorts.

Course of action	Therapy	Train cohort N (%)	Test cohort N (%)
A (upfront surgery)	Cisplatin-based chemotherapy Adjuvant osimertinib Adjuvant therapy No adjuvant therapy	17 (63.0%) 0 (0.0%) 0 (0.0%) 10 (37.0%)	3 (37.5%) 2 (25.0%) 3 (37.5%) 0 (0.0%)
B (neoadj+surgery)	Platinum-based chemotherapy Platinum-based chemotherapy +durvalumab/placebo (AEGEAN (36)) Platinum-based chemotherapy +atezolizumab/placebo (IMpower030 (35))	17 (89.5%) 1 (5.3%) 1 (5.3%)	1 (33.3%) 2 (66.7%) 0 (0.0%)
C (CRT)	Platinum-based chemotherapy Platinum-based chemotherapy + durvalumab/placebo	8 (42.1%) 11 (57.9%)	3 (37.5%) 5 (62.5%)

N, number; Neoadj, neoadjuvant; CRT, chemoradiotherapy.

### 3.2 First treatment course classification: Course A versus Rest (B+C)

For the first classification task, 13 features were selected by the final model as most informative for distinguishing course A from the Rest. These included four clinicopathological features (smoking status and mediastinal LN stage), and nine radiomic features (four from the primary tumor and five from mediastinal LNs). Lesion-derived features captured density, heterogeneity, intensity uniformity, and spatial distribution, whereas LN-derived features reflected morphological complexity, intensity variability, and malignancy suspicion (LUNG-RADS).

**Figure 3:**
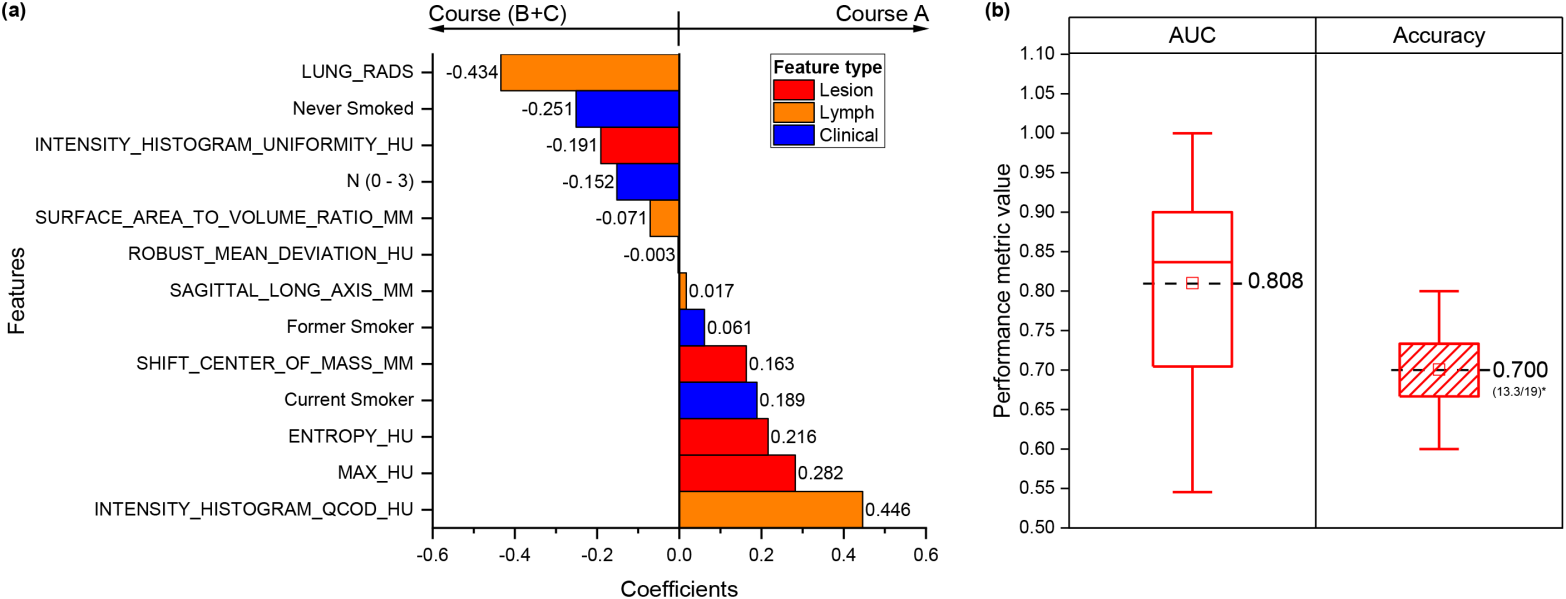
First treatment course classification: course A (upfront surgery) versus Rest (courses B+C). **(a)** Bar plot of the signed coefficients for features selected by the final model. The plot shows the magnitude and direction of each feature’s contribution and indicates whether the feature originates from the primary lesion, mediastinal lymph node, or clinical variables. Positive coefficients increase the likelihood of assignment to Course A, while negative coefficients favor multimodality treatment (Courses B+C). **(b)** Model performance in the test cohort, shown as distributions of AUC and accuracy across 100 bootstrap resamplings. The number of correctly classified patients is reported below the accuracy value. Abbreviation: AUC, area under the curve.

Interpretability was enhanced using a bar plot of the signed model coefficients (**Figure 3a**), which illustrates both the magnitude and direction of each feature’s contribution and identifies whether features originate from the primary tumor, lymph nodes, or clinical data. Positive coefficients increased the likelihood of assignment to upfront surgery (Course A), while negative coefficients favor multimodality treatment (Courses B+C). These patterns highlight a coherent clinico-radiomic profile associated with surgical selection.

The model achieved an AUC of 0.847 (95% CI: 0.615 – 1.000) and accuracy of 0.795 (95% CI: 0.600 – 0.937) in the train cohort. Performance remained consistent in the test cohort, with an AUC of 0.808 (95% CI: 0.600–1.000) and accuracy of 0.700 (95% CI: 0.533–0.902) (**Figure 3b**). Features retained in the final model corresponded to those with the highest recurrence across resampling iterations, supporting their stability (see **Supplementary Material**).

### 3.3 Second treatment course classification: Course B vs. C

For the second classification task, nine features were selected: four clinicopathological (age, tumor size category, mediastinal LN stage, and platelet count) and five radiomic features (three from the primary tumor and two from LNs). Tumor-related features described elongation, percentage ground-glass opacity, and calcifications, while LN features included sphericity and intensity variability (RMS).

**Figure 4:**
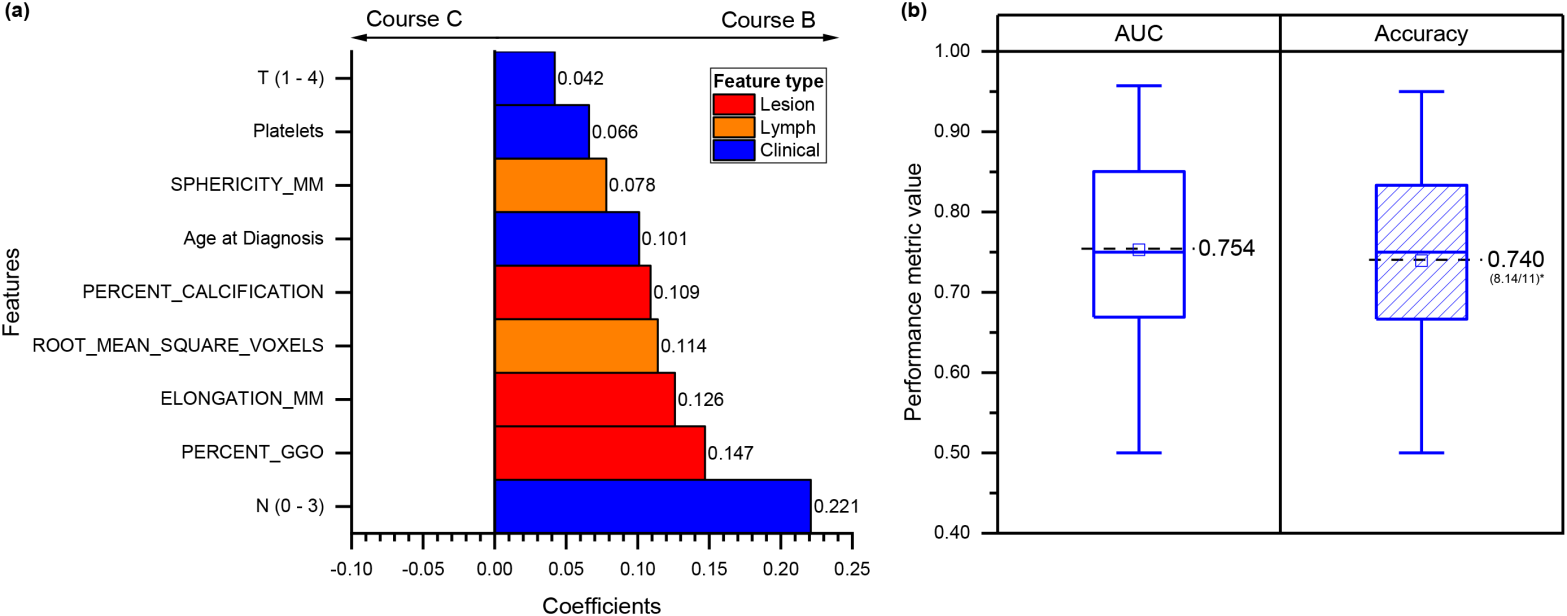
Second treatment course classification: course B (neoadjuvant therapy followed by surgery) versus course C (chemo-radiotherapy). **(a)** Bar plot of the signed coefficients for features selected by the final model. The plot depicts the magnitude of each feature’s contribution and specifies whether it originates from the primary lesion, mediastinal lymph node, or clinical variables. Positive coefficients indicate increasing likelihood of assignment to Course B relative to Course C. **(b)** Model performance in the test cohort, shown as distributions of AUC and accuracy across 100 bootstrap resamplings. The number of correctly classified patients is reported below the accuracy value. AUC, area under the curve.

A signed-coefficient bar plot was generated for the final model (**Figure 4a**). In this task, all coefficients were positive, indicating that higher feature values were consistently associated with allocation to Course B (neoadjuvant therapy followed by surgery). The plot highlights relative importance rather than directional contrast and provides a clear visualization of how clinical, primary-tumor, and LN-derived radiomic descriptors contribute to discrimination.

The model achieved an AUC of 0.740 (95% CI: 0.514 – 0.990) and accuracy of 0.700 (95% CI: 0.540 – 0.900) in the train cohort. Test performance was comparable, with an AUC of 0.754 (95% CI: 0.500–0.980) and accuracy of 0.740 (95% CI: 0.500–0.960) (**Figure 4b**). As with the first classifier, the features selected for the final model were among those with the highest recurrence frequencies across validation iterations, supporting their robustness **Supplementary Material**.

### 3.4 Survival outcomes

Although MTB decisions were used as the reference standard, survival curves were included to demonstrate clinical consistency.

**Figure 5:**
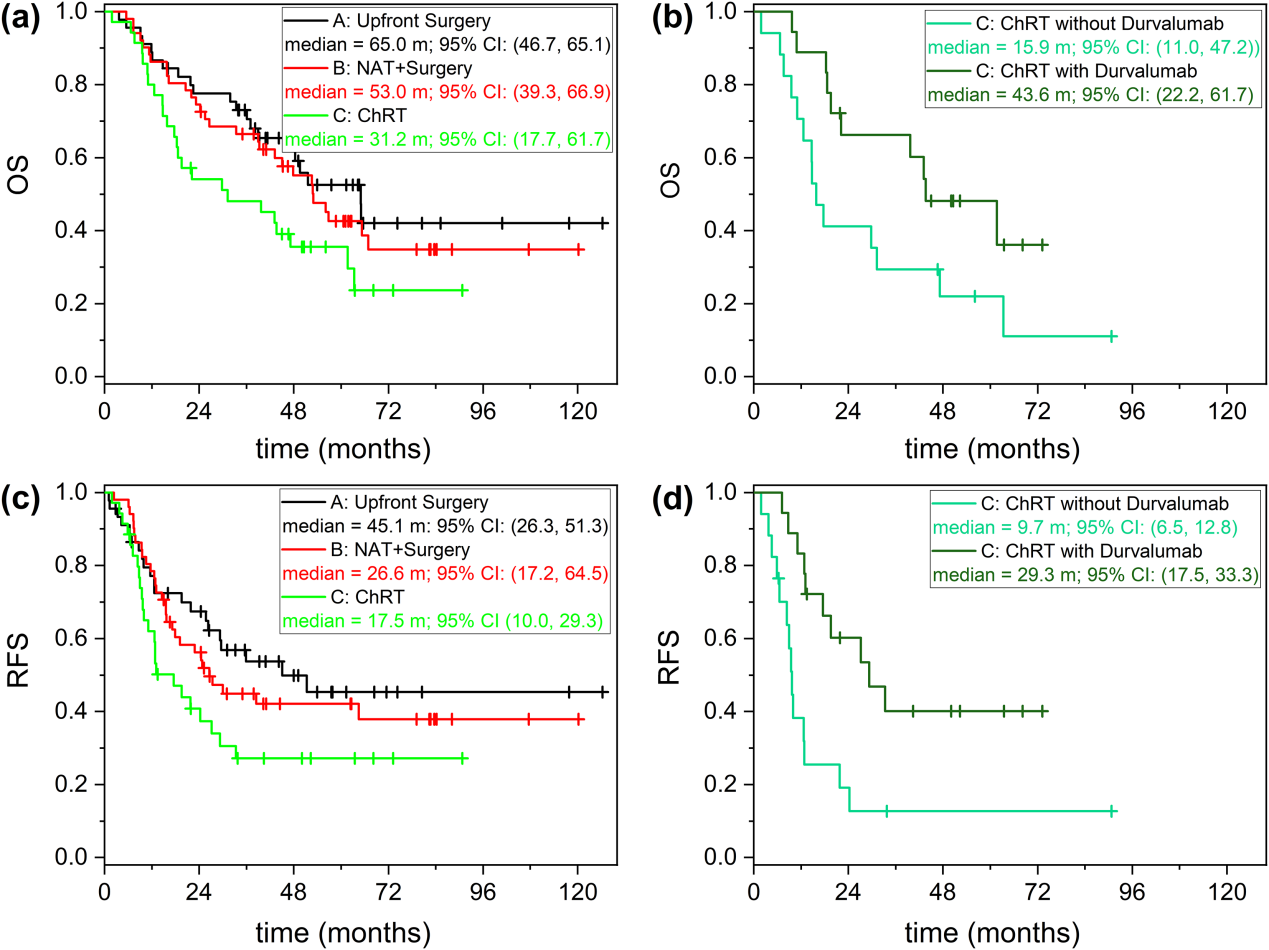
Survival curves illustrating clinical consistency of multidisciplinary tumor board decisions. **(a)** Overall survival (OS) by treatment course: A (upfront surgery), B (neoadjuvant therapy + surgery), C (chemo-radiotherapy); **(b)** OS in course C by receipt of consolidation durvalumab. **(c)** Relapse-free survival (RFS) by treatment course; **(d)** RFS in course C by receipt of consolidation durvalumab. ChRT, chemo-radiotherapy; NAT, neoadjuvant therapy; OS, overall survival; RFS, relapse-free survival.

In the ITT train population (N = 131), with median follow-up of 63.6 months, median RFS and OS were 27.2 months (95% CI: 19.6–45.1) and 51.6 months (95% CI: 43.1–65.0), respectively. Median OS was 65.0, 53.0, and 31.2 months for courses A, B, and C, respectively, while median RFS was 45.1, 26.6, and 17.5 months. For patients in course C, consolidation durvalumab was associated with improved OS (43.6 vs. 15.9 months) and RFS (29.3 vs. 9.7 months) (**Figure 5**). These curves support the clinical relevance of MTB treatment choices.

A multi-parametric linear regression analysis was performed to investigate the association of selected clinicopatological and radiomic features with OS and RFS.

In the course A versus rest classification, LUNG_RADS_Lymph and INTENSITY_HISTOGRAM_ROBUST_MEAN_DEVIATION_HU of the primary lesion were significantly correlated with OS (p=0.0306) and RFS (p = 0.0050), respectively (R2-adj = 0.09083). In the course B versus C classification, ROOT_MEAN_SQUARE_VOXELS_Lymph was significantly associated with OS (p=0.0003; R2-adj=0.1492) and RFS (p=0.0136; R2-adj= 0.06797).

These results suggest that certain radiomic features may carry prognostic information beyond their predictive value for treatment choice, complementing the interpretability analyses provided by the signed-coefficient visualizations.

## 4 Discussion

Optimizing treatment strategies for LA-NSCLC remains a major clinical challenge, as decisions must balance resectability, patient fitness, and evolving systemic therapies within a multidisciplinary framework (37). Determining surgical eligibility is particularly complex, with tumor size and lymph node involvement serving as critical (38, 39). In resectable disease, perioperative strategies are increasingly characterized by the availability of immunotherapy and targeted agents (40). In unresectable cases, consolidation immunotherapy after concurrent chemoradiation has significantly improved survival and shifted real-world treatment patterns (22, 32, 41). More recently, neoadjuvant and perioperative chemo-immunotherapy have shown high pathological complete response rates, further blurring the boundaries of resectability (42–44). These advances raise new questions, including how best to identify patients benefiting from adjuvant or consolidation immunotherapy beyond PDL1 expression levels (45) and how long adjuvant immunotherapy after radical surgery should be administered (46).

In this context, the role of a multidisciplinary tumor board is critical, particularly in borderline cases (47). A high-volume, experienced MTB including oncologists, radiation oncologists, thoracic surgeons and pulmonologists, interventional radiologists, nuclear medicine physicians, pathologists, and molecular biologists, is essential for evidence-based decision-making (48). However, the availability of such expertise is heterogeneous, and hub-and-spoke models are often required (49). Tools that integrate clinical and imaging data, such as radiomics, may help refine MTB decisions and support treatment personalization.

Our proof-of-concept study demonstrates the feasibility of using an AI-based radiomics pipeline (RadiomiX) (34) to classify stage III NSCLC patients into three therapeutic groups: upfront surgery, neoadjuvant therapy followed by surgery, or CRT. The models integrated both clinical variables and radiomic features from primary tumors and LNs, with LN-derived features providing complementary and clinically relevant information. Classification performance was robust in both training and test cohorts, supporting the potential role of this approach in real-world decision-making. In addition to overall performance, we assessed feature stability across the full modeling workflow and observed a high recurrence of top-ranked features across cross-validation iterations. This consistency supports the robustness of the radiomic and clinical signatures identified. To enhance interpretability, we also examined the direction and magnitude of feature influence using signed model coefficients, which provided an intuitive representation of the clinico-radiomic phenotype associated with each therapeutic pathway.

While survival analysis was not a primary endpoint, the alignment of survival curves with MTB recommendations provides reassurance that treatment allocation was clinically appropriate and consistent with published benchmarks (7–15, 20, 21, 32). Among selected clinical variables, age, smoking status, platelet levels, and LN stage influenced treatment stratification, in agreement with previous reports linking these factors to treatment tolerance, immune response, and prognosis (50–53). Radiomics added quantitative imaging biomarkers that complemented standard staging, which is subject to limitations such as false-negative or false-positive results in endoscopic or metabolic LN assessment (33, 53). Compared with earlier attempts to integrate AI into MTB workflows (e.g., in hepatocellular carcinoma (54)), our model achieved higher accuracy, likely due to the combination of clinical and radiomic features rather than clinical data alone.

The main limitations of our study are the modest sample size, retrospective design, and heterogeneity of imaging protocols, which led to the exclusion of several patients. Recruitment of stage III NSCLC is inherently challenging, as this group represents only about one-third of all lung cancer cases.

Evolving standards of care during the study period (2012–2022) also constrained the integration of molecular features, such as EGFR or ALK status, which are now routinely incorporated into treatment decision-making (18–20). Access to neoadjuvant or perioperative chemo-immunotherapy was similarly limited to clinical trials during most of the study timeframe. These factors emphasize the exploratory and hypothesis-generating nature of our findings. Another limitation concerns model interpretability. Inter-observer variability in lesion segmentation is a recognized source of uncertainty in radiomics. Although consensus and senior review were employed to minimize variability, no formal ICC-based reproducibility analysis was performed. Future work will include robustness assessment according to Zwanenburg et al. (55).

While the signed-coefficient analysis offered insight into the contribution of individual features, more advanced explainability frameworks (e.g., SHAP) were not implemented due to the exploratory nature and sample size of this study. Future work will incorporate model-agnostic explainability methods to better characterize radiomics-derived phenotypes and to support transparent clinical translation.

Future validation in prospective, multicenter settings will be essential to confirm these results under standardized imaging protocols, updated staging, and comprehensive molecular profiling. Such studies will determine whether integrated clinico-radiomic models can reliably support MTB decisions and improve treatment personalization.

## 5 Conclusions

This study provides initial evidence that AI-based radiomics can support MTB decision-making in stage III NSCLC. By integrating clinical and imaging-derived features, our models achieved consistent classification performance across major therapeutic pathways. Although limited by its retrospective design and sample size, this proof-of-concept work highlights the feasibility of radiomics-driven decision support and provides the rationale for prospective validation in treatment personalizing for LA-NSCLC.

## Data Availability

The original contributions presented in the study are included in the article/**Supplementary Material**. Further inquiries can be directed to the corresponding author.
