# Supplementary material for: Radiomic approach to support multidisciplinary tumor board decision-making in locally advanced non-small cell lung cancer
Source: Front Oncol. 2025 Dec 19;15:1713847. doi: 10.3389/fonc.2025.1713847 (PMC12757298; doi:10.3389/fonc.2025.1713847)
Supplement: Supplementary file 1 [file DataSheet1.docx]

Supplementary Material

# Supplementary Data

**1.1** **First treatment course classification: A (upfront surgery) versus Rest (B+C) details**

Oversampling method: SMOTE (60, 35)

Scaler: StandardScaler

Power transformation: True

Classifier: SVM

Hyperparams: C=0.1, kernel=linear

Feature selection method: MRMR

Classification mode: One vs Rest

AUC: 0.847 (95% CI: 0.615 – 1.000)

Accuracy: 0.795 (95% CI: 0.600 – 0.937).

F1: 0.701

External test AUC: 0.808 (95% CI: 0.600 – 1.000)

External test Accuracy:0.700 (95% CI: 0.533 – 0.902)

**1.2 Second treatment course classification: induction therapy followed by surgery (B) versus patients eligible for CRT (C)**

Oversampling method: RandomOversampler (30, 26)

Scaler: StandardScaler

Power transformation: True

Classifier: Random Forest

Hyperparams: Criterion = Entropy; Max Depth = 8; Max features = log2; n_estimators = 9, bootstrap = FALSE

Feature selection method: MRMR

Classification mode: One vs one

AUC: 0.740 (95% CI: 0.514 – 0.990)

Accuracy: 0.700 (95% CI: 0.540 – 0.900)

F1: 0.585

External test AUC: 0.754 (95% CI: 0.500 – 0.980)

External test Accuracy: 0.740 (95% CI: 0.500 – 0.960)

# Supplementary Figures and Tables

##
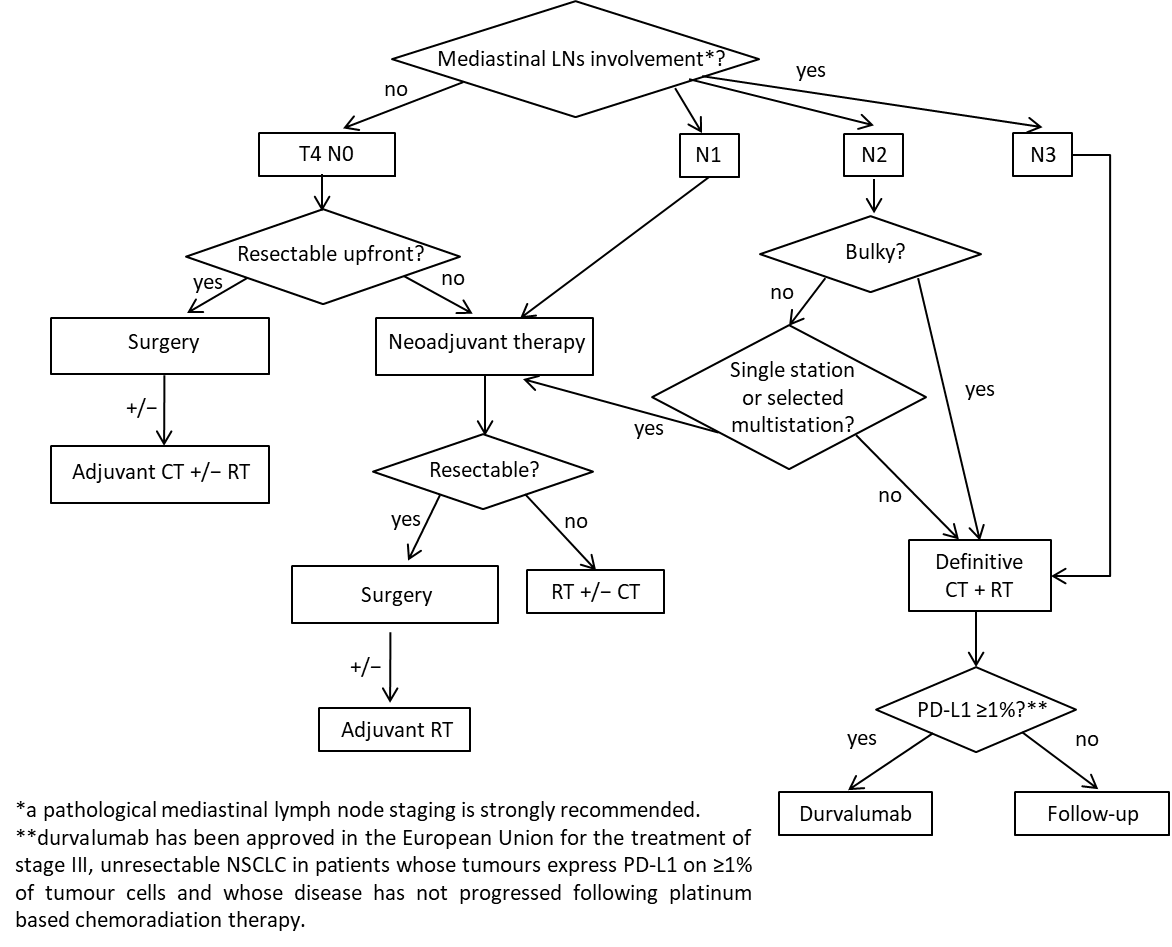
 Supplementary Figures

**Supplementary Figure 1.** Management of stage III NSCLC based on imaging, invasive lymph node staging and multidisciplinary assessment.

Abbreviations: CT, chemotherapy; LN, lymph node; NSCLC, non-small cell lung cancer; RT,radiotherapy.


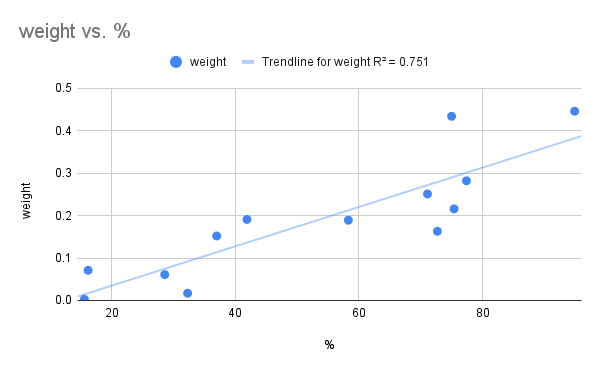


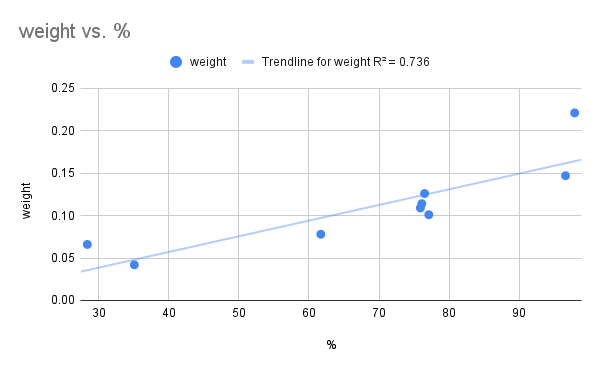
**Supplementary Figure 2.** Relationship between feature recurrence and coefficient magnitude in the A vs. Rest classification model. Each point represents a feature, with the x-axis showing the percentage of cross-validation iterations in which the feature was selected (% recurrence) and the y-axis showing the absolute value of its coefficient in the final model (weight). The plot illustrates that features consistently selected across iterations tend to have higher influence on model predictions.

**Supplementary Figure 3.** Relationship between feature recurrence and coefficient magnitude in the B vs. C classification model. Each point represents a feature, with the x-axis indicating the percentage of cross-validation iterations in which the feature was selected (% recurrence) and the y-axis showing the absolute value of its coefficient in the final model (weight). This visualization highlights the link between selection frequency and feature contribution to treatment assignment.

**Supplementary Tables**

**Supplementary Table 1.** Recurrence and relative contribution of features in the A vs. Rest classification model. For each feature, the table reports the percentage of cross-validation iterations in which it was selected (%), the absolute number of iterations (N), and the absolute value of the corresponding model coefficient (weight) in the final classifier, reflecting the feature’s influence on treatment assignment.

| A vs. Rest | % | N | weight |
| --- | --- | --- | --- |
| ROBUST_MEAN_DEVIATION_HU | 15.6 | 8 | 0.003 |
| SURFACE_AREA_TO_VOLUME_RATIO_MM | 16.2 | 8 | 0.071 |
| Former Smoker | 28.6 | 14 | 0.061 |
| SAGITTAL_LONG_AXIS_MM | 32.3 | 16 | 0.017 |
| N (0 - 3) | 37.0 | 19 | 0.152 |
| INTENSITY_HISTOGRAM_UNIFORMITY_HU | 41.9 | 21 | 0.191 |
| Current Smoker | 58.3 | 29 | 0.189 |
| Never Smoked | 71.1 | 36 | 0.251 |
| SHIFT_CENTER_OF_MASS_MM | 72.7 | 36 | 0.163 |
| LUNG_RADS | 75.0 | 38 | 0.434 |
| ENTROPY_HU | 75.4 | 38 | 0.216 |
| MAX_HU | 77.4 | 39 | 0.282 |
| INTENSITY_HISTOGRAM_QCOD_HU | 94.9 | 47 | 0.446 |

**Supplementary Table 2.** Recurrence and relative contribution of features in the B vs. C classification model. For each feature, the table reports the percentage of cross-validation iterations in which it was selected (%), the absolute number of iterations (N), and the absolute value of the corresponding model coefficient (weight) in the final classifier, reflecting the feature’s influence on treatment assignment.

| B vs. C | % | N | weight |
| --- | --- | --- | --- |
| N (0 - 3) | 97.9 | 49 | 0.221 |
| PERCENT_GGO | 96.6 | 48 | 0.147 |
| ELONGATION_MM | 76.5 | 38 | 0.126 |
| ROOT_MEAN_SQUARE_VOXELS | 76.1 | 38 | 0.114 |
| PERCENT_CALCIFICATION | 75.9 | 38 | 0.109 |
| Age at Diagnosis | 77.1 | 39 | 0.101 |
| SPHERICITY_MM | 61.7 | 31 | 0.078 |
| Platelets | 28.4 | 14 | 0.066 |
| T (1 - 4) | 35.1 | 18 | 0.042 |
